# The effectiveness of a comprehensive corrective exercises program and subsequent detraining on alignment, muscle activation, and movement pattern in men with upper crossed syndrome: protocol for a parallel-group randomized controlled trial

**DOI:** 10.1186/s13063-020-4159-9

**Published:** 2020-03-12

**Authors:** Mohammad Bayattork, Foad Seidi, Hooman Minoonejad, Lars Louis Andersen, Phil Page

**Affiliations:** 1 Health and Sports Medicine Department, Faculty of Physical Education and Sport Sciences, University of Tehran, Tehran, Iran; 2 Sport Sciences and Physical Education, Faculty of Humanities Science, University of Hormozgan, Bandar Abbas, Iran; 3 National Research Centre for the Working Environment, Copenhagen, Denmark; 4 Sport Sciences, Department of Health Science and Technology, Aalborg University, DK-9220 Aalborg, Denmark; 5 Performance Health, Baton Rouge, Louisiana USA

**Keywords:** Corrective exercises, Alignment, Muscle activation, Movement pattern, UCS

## Abstract

**Background:**

Upper crossed syndrome (UCS) refers to specific altered muscle activation and changed movement patterns along with some postural deviations in the upper quarter of the body. This syndrome might contribute to the dysfunction of the cervicothoracic and glenohumeral joints.

**Objectives:**

The present study will aim to investigate the effectiveness of a comprehensive corrective exercises program (CCEP) and subsequent detraining on alignment, muscle activation and movement pattern in men with UCS.

**Methods/design:**

This is a parallel-group randomized controlled trial. Participants will be 22 men aged 18 to 28 years who are suffering from UCS. Participants in the intervention group will conduct CCEP (three times a week for 8 weeks), followed by 4 weeks of detraining. The control group will do their daily activities. Participants will be randomized (1:1) into the intervention or the control group. The primary outcome will be upper trapezius activations. Secondary outcomes consist of electromyography of middle and lower trapezius and serratus anterior muscles, scapular dyskinesis test, forward head and shoulder angles, thoracic kyphosis angle, and neck flexion pattern test.

**Discussion:**

We propose to evaluate the effectiveness of a randomized controlled trial of a CCEP in men with UCS on their alignment, selected muscle activations, and relevant movement patterns. Results from our trial may provide new insights into the effects of exercise not only on the alignment but also on muscle activation and movement patterns that are important outcomes for people with postural malalignments and, if successful, could assist therapists in evidence-based clinical decision-making.

**Trial registration:**

Iranian Registry of Clinical Trials, IRCT20181004041232N1. Registered on 26 October 2018.

## Background

Most people will see a medical practitioner or another health care provider at least once in their lifetime because of neck, shoulder and back pain [1, 2]. At a societal level, these pains are also responsible for substantial costs, including healthcare expenditure, disability insurance, and work absenteeism [3]. Previous studies showed that these pains might be associated with abnormal alignments [4, 5]. One of these malalignments is the upper crossed syndrome (UCS), which was defined as a muscular imbalance pattern by Vladimir Janda MD (1923–2002) [6]. UCS refers to specific altered muscle activation and movement patterns along with some postural deviations [7]. Alterations in muscle activation include overactivity of the upper trapezius, levator scapula, and pectorals muscles and underactivity of the deep cervical flexors, middle and lower trapezius, and serratus anterior [8]. Due to kinetic and muscular chains, there are altered scapular movement patterns (scapular dyskinesis) and specific postural changes, including forward head and shoulder posture and increased thoracic kyphosis [7, 9]. These changes can lead to reduction in the stability of the glenohumeral joint and to various musculoskeletal symptoms in the head, neck, and shoulder [7, 8, 10].

Over recent decades, therapists have been seeking to design appropriate exercises to correct musculoskeletal malalignments mainly through structural and functional approaches [11–13]. In the traditional structural approach, the changes observed in malalignments such as in UCS are attributed to biomechanics and are presumed to lead to adjustments in the length and strength of local muscles [11, 14]. This may account for the stretching of short muscles and strengthening of weakened muscles at the site of the problem in the correction phase, while ignoring other related malalignments [14]. Interestingly, despite the popularity of this method, very little research has been conducted based on this theory [15]. Furthermore, some review studies have questioned the effectiveness of strengthening and stretching exercises to improve postural disorders [16, 17].

In contrast, the functional (neurological) approach to musculoskeletal problems is based on the interaction of the central and peripheral nervous systems, and the involvement of the muscular and skeletal structures in producing and controlling motion [6, 18, 19]. In this functional approach, the musculoskeletal problems are attributed to the role of muscles in motor function; furthermore, changes in the alignment result not only from changes in muscle length and strength but also from more important changes in muscle neuromuscular factors, such as muscle recruitments [13, 20]. In fact, the motor control unit may change the muscle activation strategy for temporary stabilization due to the presence of dysfunction. These changes in motor recruitment will alter the muscular balance, movement patterns, and eventually the motor program [12]. Similarly, Hodges et al. noted that motor control interventions require tailoring to each individual’s posture, muscle activation, and movement pattern [21]. However, this theory has not been tested in practice for the prevention and treatment of musculoskeletal malalignments.

Hence, for the assessment of UCS, the alignment and its side effects are often evaluated, such as increase in thoracic kyphosis or forward head angles, while less attention has been paid to the keystone, i.e., the scapulae, and the relevant altered muscle activation and movement patterns [22]. In this regard, many researchers and therapists have only evaluated one of the affected regions, such as head, shoulders, or spine, separately and reported a degree of postural deviation regardless of other relevant malalignments and patterns of the muscle activation and related movement patterns, such as scapulohumeral rhythm or neck flexion [23–25]. In addition, the design and implementation of the training protocol are based on the traditional structural approach, in which stretching exercises for short muscles and strengthening exercises for weak muscles are prescribed at the site of malalignment [22, 24], while the neuromuscular factors and related movement patterns may not be considered. To the best of our knowledge, no studies identifying and correcting UCS have considered the three components of alignment, movement pattern, and muscle activity in both assessment and correction processes.

### Study objectives

The primary aim of the present study is to evaluate the effectiveness of a comprehensive corrective exercise program (CCEP) in young men with UCS for 8 weeks, as measured by alignment (position of the scapula, head and neck, shoulder, and thoracic spine), electromyography activity of selected muscles (upper, middle and lower trapezius, serratus anterior), and specific movement patterns (scapulohumeral rhythm and neck flexion). A secondary aim is to evaluate the effects of the program after 4 weeks of detraining after the intervention.

## Methods/design

### Study design

**Fig. 1:**
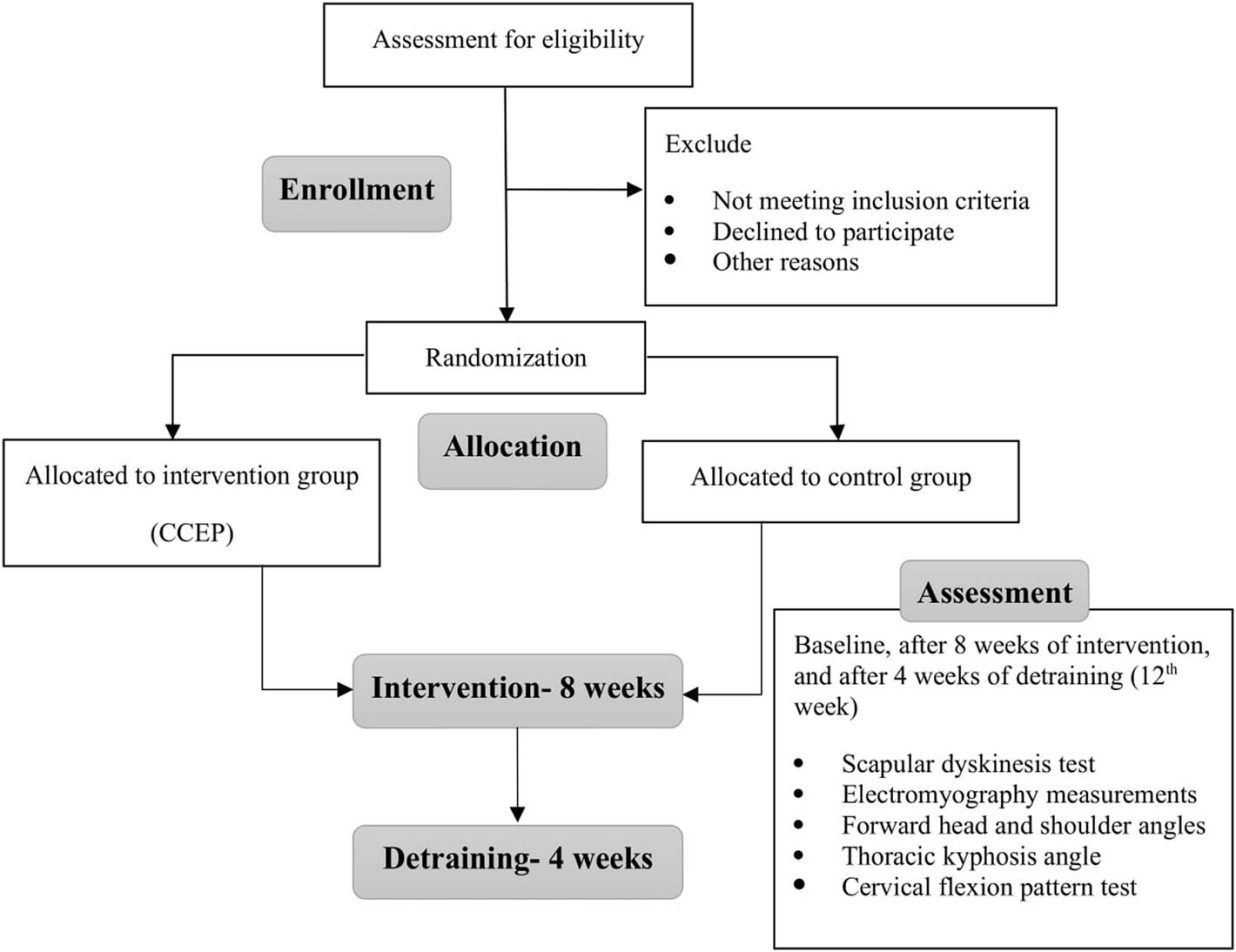
Study flowchart

This is a parallel-group randomized controlled trial comparing an intervention group receiving an 8-week CCEP followed by 4 weeks of detraining to a control group who will only do their daily activities. The study will be performed at the Laboratory of Health and Sports Medicine Department, University of Tehran, Tehran, Iran. Initially, participants will take part in the baseline assessment process. They will then receive the intervention for 8 weeks. After the end of the intervention phase, all the measurements will be repeated. Finally, a follow-up assessment will be performed after a 4-week detraining period. The study schedule is presented in Table 1 and a flow diagram is shown in Fig. 1. The study protocol is reported in accordance with the SPIRIT guideline.

**Table 1:**
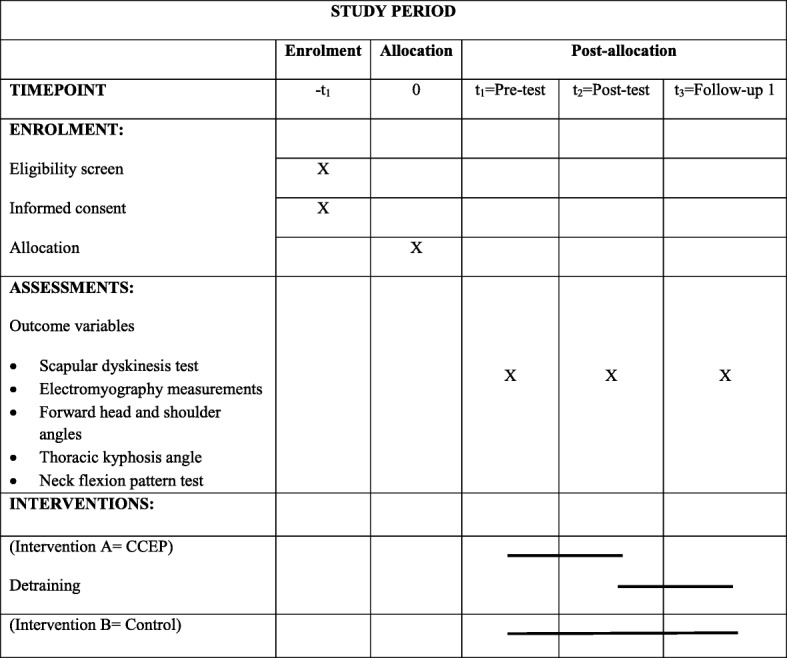
Schedule of the study

### Ethical aspects

Before starting the project, all participants will be asked to complete and sign an informed consent form. Ethics approval was obtained on August 28, 2017, from the Ethics Committee on Research at University of Tehran, Iran (IR.UT.REC.1395026). The protocol was approved at the Iranian Registry of Clinical Trials on 2018-10-26 (IRCT20181004041232N1).

### Study participants and eligibility criteria

The participants consist of 24 men with UCS aged 18 to 28 years. They will be recruited from the students of the University of Tehran, Iran, through advertisements on bulletin boards. They will be screened primarily by observation for three main factors related to UCS, including altered alignment, muscle activation, and movement patterns. Since the scapulae are the keystone in UCS, participants who have any abnormality in the position and rhythm of the scapula, as measured by the scapular dyskinesis test, will be recruited. In addition, they will be assessed for presenting any postural changes such as forward head (≥ 44°), round shoulder (≥ 49°), or excessive thoracic kyphosis (≥ 42°) as measured by photogrammetry and flexicurve, respectively [15]. Also, to ensure any change in muscle activation is related to postural changes, some confirmatory tests, including muscle length tests for upper trapezius and pectoral muscles as well as muscle strength tests for middle and lower trapezius and deep cervical flexor, will be used. Individuals will be excluded from the research process if they do extra physical activity and sports that may affect the outcomes of the research, have any visible malalignment in the pelvis and lower extremities, have a history of fracture, surgery, or joint diseases in the spine, shoulder and pelvis, have a rotation greater than 5° on the forward bending test because of scoliosis [15], or have a bodyweight outside the normal range (body mass index between 18 and 25) [23].

### Randomization

Participants will be randomized using computer-generated block randomization in a 1:1 ratio, followed by a concealed allocation through opening sequentially numbered, opaque and sealed envelopes; a card inside will indicate the group into which the participant will randomly be allocated, i.e., the intervention or the control group. Participants can discontinue the project at any time. However, all efforts will be made to avoid missing data. The specific way to deal with missing data will be determined at a data review meeting before starting statistical analyses.

### Comprehensive approach

**Fig. 2:**
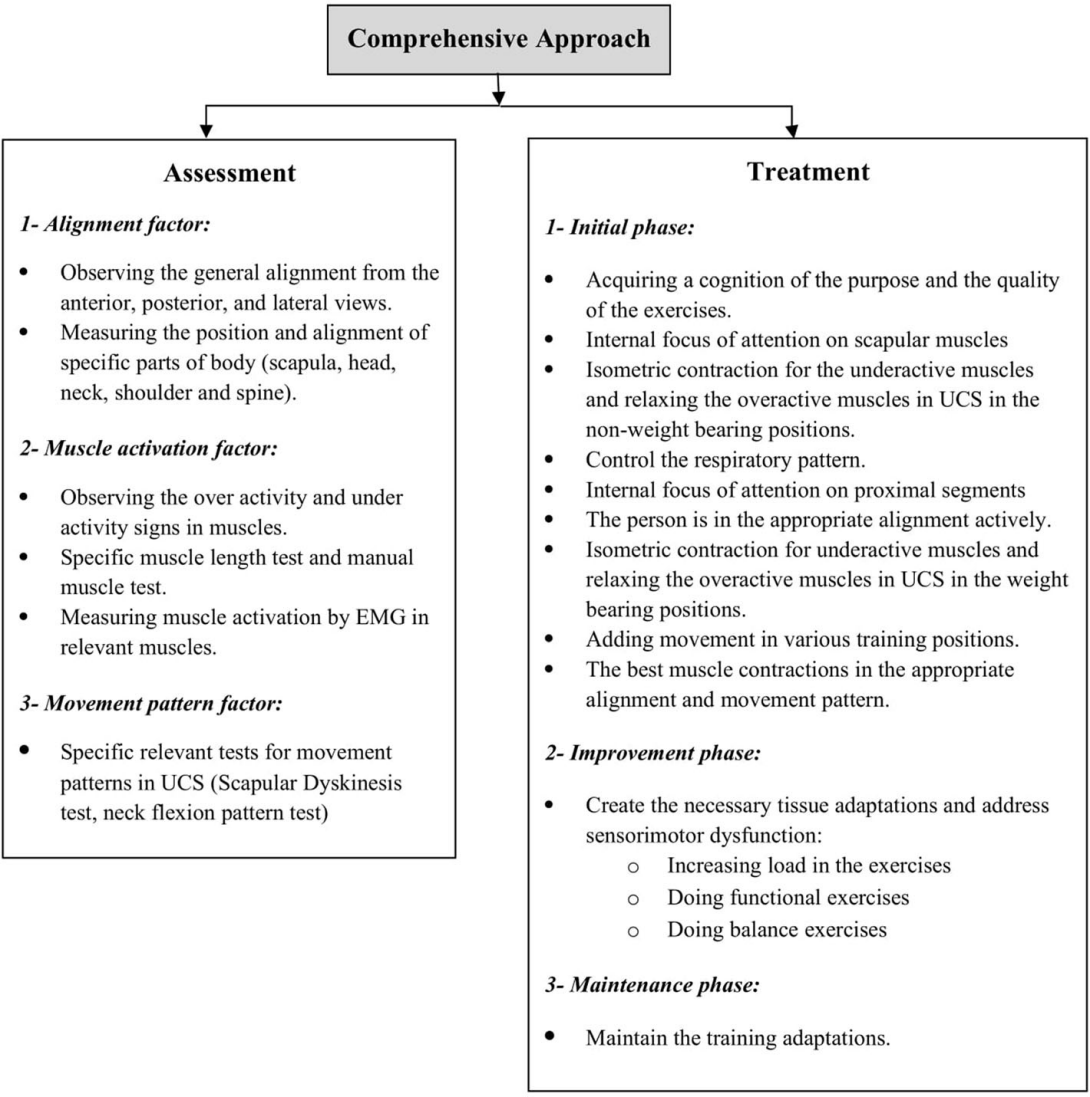
Comprehensive approach flowchart

The comprehensive approach (Fig. 2), which is a new approach to corrective exercises [15], takes advantage of the strengths and weaknesses of traditional approaches to achieve the best outcome in correcting musculoskeletal malalignments. It was initiated in 2014 by Seidi et al., who compared the efficacy of comprehensive and traditional corrective exercise programs on the kyphosis angle [15]. The general purpose of this approach is to pay attention to alignment, muscle activation, and movement pattern simultaneously across the whole body rather than at just a single site during both the assessment and correction phases.

#### Intervention

An 8-week CCEP will be structured to include three phases. In the initial phase of the exercise program, the participants will become cognizant of the purpose and the quality of the exercises. This is characterized as voluntary control of exercise, requiring cortical regulation of movement and a lot of concentration on the part of the participants [12]. Participants will be asked to focus only on the scapular muscles, i.e., an internal focus [26] while their alignment is corrected passively. So, the exercises will be executed in non-weight bearing positions and the participants only try to contract underactive muscles isometrically and relax overactive muscles around the scapula for normalization of scapular position and motion [27, 28]. The participants will be instructed to reproduce this orientation of scapula actively using auditory (from therapist) and kinesthetic cues such as palpation [28]. Once a participant regains sufficient control over scapular muscles, he will focus externally on it and turn the internal focus of attention to correcting proximal segments through chin tuck, retraction of shoulders, and straightening the upper thoracic spine [29]. Then, he will do the exercises in different weight-bearing positions, and after restoring muscle balance in the static conditions, he will try to add upper extremity movements in various training positions. Also, the respiratory pattern of participants will be controlled during this phase and necessary feedback will be provided [13]. Since the quality of exercises is highly important in this phase, the participants should not feel fatigued while doing the exercises because fatigue may alter optimal muscle activations and movement patterns [30].

Once the participants can contract the appropriate muscles in a correct alignment during the best movement pattern, they should be able to maintain this for a long time. This goal will be addressed in the improvement phase when necessary tissue adaptations occur by increasing the load of exercises [31, 32]. Placing the participants in weight-bearing positions and using some tools such as training balls and Thera-Bands will reinforce their abilities gradually. During this phase, the participants will only focus on the goal of exercises (external focus) [29]. Moreover, because UCS is representative of underlying potential sensorimotor dysfunction, some functional balance exercises will be included [12]. The frequency and intensity of the exercises will increase progressively during the study, provided that participants can demonstrate good-quality movement. In the final phase, i.e., the maintenance phase, the participants will try to maintain the training adaptations [32]. All exercises will be performed under the supervision of a corrective exercise specialist. The participants will not conduct any extra exercises at home. However, they will be asked to avoid sustaining poor posture. More details about the intervention protocol are presented in Appendix 1. The control group will be asked to do their ordinary daily activities and not to participate in any exercise programs. After the study is completed, the control group will undergo the exercise intervention protocol for ethical reasons.

### Outcome measures

All outcome measurements will be performed by the main researcher at baseline, 8 weeks (after intervention), and 12 weeks (follow-up). Demographic information (i.e., sex, age, body mass index) will be measured before the intervention.

#### Outcome measures

Upper trapezius activation (measured with surface electromyography) is the primary outcome measure. Secondary outcomes include electromyography measurements of middle and lower trapezius and serratus anterior muscles, scapular dyskinesis, forward head and shoulder angles, thoracic kyphosis angle, and neck flexion pattern.

##### Electromyography measurements

Surface electromyography of the scapular stabilizer muscles (including upper, middle, lower trapezius, and serratus anterior) will be performed using an ME-6000 Megawin. The participants will execute humeral abduction without resistance in three phases (concentric, isometric, and eccentric) lasting for 3 s each. They will already have been trained to move correctly and at the right speed so that they can perform the movement five times; rest time is 3 s between movements. Disposable Ag-AgCl electrodes with a diameter of 2 cm and a 2 cm spacing between two poles of electrodes will be used and data will be recorded at a frequency of 1000 Hz. The location of the electrodes will be determined using the SENIAM protocol and based on valid scientific papers [33, 34]. The maximum voluntary isometric contraction (MVIC) will be recorded to normalize the signals. More details about the location of the electrodes and MVIC positions are presented in Appendix 2. The data from the mean square root (RMS) will be used in the process of measuring muscle activation. To determine the onset of muscle activity, only the concentric phase of the motion will be used and it will be based on the onset of the deltoid muscle. Moreover, the onset of the activity will be from the point where the level of muscle activity reaches two standard deviations above the rest of the muscle activity [34].

##### Scapular dyskinesis

The current recommendation for clinical assessment based on a prior consensus meeting is the use of the dynamic scapular dyskinesis test according to the procedure described by McClure et al. [35]. The position and motion of scapula are characterized by dyskinesis as a “yes” (presence of deviation or dysrhythmia/asymmetry bilaterally) or “no” (no presence). This method has been shown to be reliable among observers and has acceptable clinical utility [35, 36].

##### Forward head and shoulder angles

The angle of the head and the shoulder will be measured using photogrammetry according to the procedure described by Seidi et al. [15]. The validity and reliability of this method have been established in previous studies [37, 38].

##### Thoracic kyphosis angle

To measure the static alignment of the thoracic spine, the flexicurve method will be used, which is a well-established, valid, and reliable technique [39, 40]. A detailed description of the procedure can be found in previous studies [15, 23].

##### Neck flexion pattern test

The participants will lie supine with knees bent. They will then be instructed to lift the head and look at their toes. Normal movement produces a smooth reversal of the normal cervical lordosis, keeping the chin tucked. Abnormal movement is compensated by the tightness of the SCM, producing an early protraction of the chin directly upward at the beginning of the motion [8].

### Sample size

The sample size was calculated using the G*Power software (G*Power, version 3.0.10, Germany). It was based on a pilot test of seven participants, and on the assumption that a 10% difference in muscle activity [34] and an 11° difference in kyphosis angle [41] between groups would constitute clinically meaningful differences. It was calculated that a sample consisting of at least 18 participants would suffice to obtain 80% power with d = 0.80 effect size, and a confidence interval of 0.95. It should be noted that the effect size was reported in the previous study which compared scapular muscle activity between the interventions and control groups. Effect sizes ranged from 0.6 to 0.9 for the EMG amplitude and onset [42]. Since a few participants may drop out of the intervention studies, we will include 24 (assuming a drop-out rate of approximately 25%).

### Statistical method and analysis

Assessments of statistical procedures will be performed using IBM SPSS version 20 for Windows (SPSS Inc., Chicago, IL, USA). All variables will be reported using the descriptive statistic (mean, standard deviation). Shapiro-Wilk test will be used to assess the normality of data. Repeated measures ANOVA will be used to compare the means. If mean difference is significant, then the Bonferroni-adjusted post-hoc test will be calculated. The independent *t*-test will also be used for comparison between groups. Finally, the effect size will be calculated for the magnitude of the difference using the Cohen method. The significance level will be set at *p* < 0.05.

## Discussion

We propose to evaluate the effectiveness of a randomized controlled trial of a CCEP in a group of men aged 18 to 28 years with UCS in terms of their alignment, selected muscle activation, and relevant movement patterns.

Clinicians believe that it is important to quantify head, shoulder, scapulae, and spinal posture behavior as they influence and are influenced by many biomechanical, motor control, and performance variables [43]. It has been assumed that exercise can correct postural malalignment, but an earlier review found little evidence to support this assumption [17]. Moreover, despite the widespread inclusion of postural correction in exercise interventions, there are limited empirical data to support its effectiveness and little is known about the most effective exercise interventions [24]. As already mentioned, it seems the most important reason is the adherence to the traditional structural approach in the previous studies. We want to conduct a randomized controlled trial based on the comprehensive approach which is adequately powered and utilizes validated outcome measurements of UCS to investigate the effects of the CCEP on both our primary and secondary outcomes. If the CCEP results in changing the alignment, muscle activation, and movement pattern, or all three, we will examine the pathways of change to determine whether changes in the alignment, muscle activation, or movement pattern can account for the change in the UCS symptoms. Furthermore, if we find out that the CCEP can improve a postural malalignment like in UCS, this evidence could enable practitioners to recommend early intervention for UCS to prevent or delay UCS-associated consequences. Therefore, various experts in the field of corrective exercises and physical therapy, equipped with the knowledge on these changes, can identify people with UCS and also adopt appropriate therapeutic strategies to correct it and prevent the occurrence of secondary consequences.

Our study has some limitations including the recruitment of only men 18 to 28 years of age. Therefore, the results of this study will not be generalizable to all people (e.g., women or men aged ≥ 28 years) with UCS. Another limitation is that this study is not a double-blind design since it is not possible, as is the case with most exercise trials.

The results of our study will be presented at scientific conferences and published in academic journals to ensure that our study will inform therapists in practice and prove beneficial to patient care. Our goal is to conduct a clinical trial that will provide therapists with evidence of the efficacy of the CCEP on the keystone and the side effects of UCS. Previous trials have often used only assessment of the alignment to investigate the improvement of a malalignment. However, we are focusing on alignment, muscle activation, and movement pattern simultaneously based on the comprehensive approach. Therefore, if our exercise intervention proves successful, our approach to improving UCS could represent a fundamental paradigm shift in exercise intervention strategies to improve postural malalignments and their consequences. Results from our trial may provide new insights into the effects of exercise not only on alignment but also on muscle activation and movement pattern, which are important outcomes for people with postural malalignments and, if successful, it could assist practitioners in individualized clinical decision-making. However, our results may have a limited transferability to all people and thus may be valid only for men.

### Trial status

This trial was registered on 2018-10-26 (registration number IRCT20181004041232N1, protocol version number 34266, https://en.irct.ir/user/trial/34266/view). The trial is currently in the stage of recruiting patients. The first patient was included on 2019-02-01. To date, ten patients have been included. The recruitment will be completed on approximately 2019-07-01.

## Figures and Tables

**Fig. 3:**
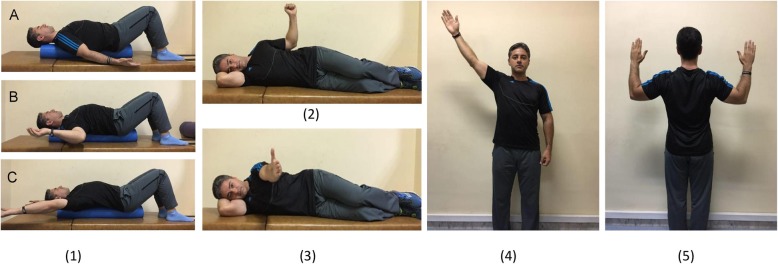
The initial phase exercises

**Fig. 4:**
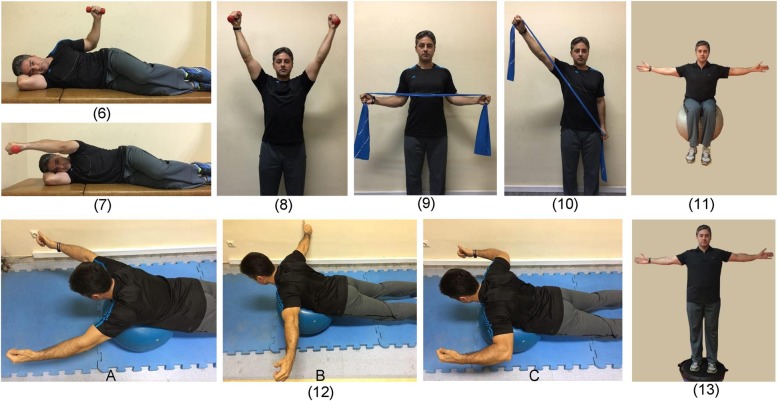
The improvement phase exercises

## Data Availability

The authors aim to make the datasets supporting the results and conclusions of this study available as supplementary files in future published articles.

## Appendix 1

### Comprehensive corrective exercise program

The duration of the exercise protocol is 8 weeks, with three sessions per week, and each session will be about an hour. Each exercise session begins with 10 min of warm-up activity and ends with 5 min of cool-down. Selected exercises are designed in three phases: initial, improvement, and maintenance [35].

#### Initial phase exercises

The initial phase exercises (Fig. 3) include laying supine on a foam roll in three different arm abduction angles (exercise 1A–C), side-lying external rotation (exercise 2), side-lying forward flexion (exercise 3), standing diagonal flexion (exercise 4), and military press (exercise 5). Participants with less ability can do exercises 4 and 5 in a sitting position. Once a participant regains muscle balance in the static conditions, he will try to add upper extremity movements in exercise positions. Exercises progress in frequency and intensity during this phase, as long as participants are able to demonstrate good quality movement. The initial phase duration is 2 weeks and the exercises will be performed for seven sets of 10-s hold to ten sets of 15-s hold.

#### Improvement phase exercises

The goal of the improvement phase is to create the necessary tissue adaptations in the participant. Therefore, during this phase Thera-Bands, weights, and training balls will be used. Improvement phase exercises (Fig. 4) include side-lying external rotation with a dumbbell (exercise 6), side-lying forward flexion with a dumbbell (exercise 7), standing diagonal flexion with a dumbbell (exercise 8), standing external rotation with Thera-band (exercise 9), standing diagonal flexion with Thera-band (exercise 10), abduction in sitting on a training ball (exercise 11), lying prone V, T, and W exercises (exercise 12), and abduction in standing on a balance board (exercise 13). Exercises are progressed by considering individual characteristics of each participant and by observing the overload principle and the progression in the number of repetitions of each set during the 4 weeks of the improvement phase. The exercises will be performed from five sets of ten repetitions to six sets of 15 repetitions.

***Maintenance phase exercises:*** The exercises are the same as in the improvement phase without any progression in intensity and frequency. The maintenance phase duration is 2 weeks.

## Appendix 2

**Table 2:** The locations of electrodes and MVIC positions

Muscle	Electrode location	MVIC position
Upper trapezius	The halfway point between the spinous process of the seventh cervical vertebra and the acromion	In a sitting position with abduction at 90°
Middle trapezius	The halfway point between the scapular medial border and the T3	In a prone position with horizontal abduction at 90° and external rotation
Lower trapezius	At 2/3 on the line from the trigonum spinea to the T8	In a prone position with horizontal abduction in 120° in line with the fibers of the muscle and the thumb pointing upward
Serratus anterior	In the mid-axillary line over the fifth rib	In a sitting position with maximum resistance given to upward rotation of the scapula with the shoulder flexed
Middle deltoid	The halfway point of the acromion and the deltoid tuberosity	In a sitting position with abduction at 90°
